# Prevalence of Possible Dementia in Patients with Maxillofacial Defects and Difficulty of Inserting Obturator in Maxillectomy Patients: Toward Better Provision of Supportive Care

**DOI:** 10.3390/jcm12072722

**Published:** 2023-04-05

**Authors:** Hongli Yu, Haruka Fujita, Masako Akiyama, Yuka I. Sumita, Noriyuki Wakabayashi

**Affiliations:** Department of Advanced Prosthodontics, Graduate School of Medical and Dental Sciences, Tokyo Medical and Dental University, Tokyo 113-8510, Japan

**Keywords:** dementia, Hasegawa’s dementia scale, defect, maxillofacial prosthetics, difficulty of inserting maxillofacial prostheses

## Abstract

As society ages, it is important to understand the prevalence of dementia and the difficulties of inserting prostheses in patients with maxillofacial defects in order to clarify issues in supportive care. We screened 183 patients for dementia using the revised Hasegawa’s dementia scale (HDS-R) at the Clinic for Maxillofacial prosthetics, Tokyo Medical and Dental University, and investigated age and sex differences in HDS-R score. We asked 47 of the 183 participants about the difficulty of inserting a maxillofacial obturator prosthesis and collected subjective comments, information about the prosthesis, and data from five assessments. Multiple regression analysis was used to reveal factors associated with insertion difficulty. Overall, 8.7% of the participants were judged to have possible dementia. Men were more likely than women to have possible dementia, and the risk increased with age. Of the 47 participants, 26 reported difficulty inserting their prosthesis, 12 of whom attributed it to their oral defect. Fourteen patients advised following doctor’s instructions to practice insertion in order to become accustomed to it. A lower HDS-R score had a significant impact on insertion difficulty. Cognitive function and difficulty inserting maxillary obturator prostheses should be considered in the provision of continuous supportive care to patients with maxillary defects.

## 1. Introduction

Maxillofacial prosthodontics provides prosthetic rehabilitation for functions such as speech, swallowing, mastication, and esthetics and is a well-recognized subspecialty of prosthodontics [1,2,3]. In 2018, Yanagi et al. conducted a study at the Clinic for Maxillofacial Prosthetics, Tokyo Medical and Dental University, and found that the average age of patients had shown an increasing trend over the previous 35 years and that almost 80% of patients had developed tumors in Japan’s now super-aging society [4], and we feel that this could be even higher now. Aging causes various problems, not only in oral function but also in manual dexterity, grip strength, cognition, and other areas [5]. In fact, the number of older adults with dementia in Japan was estimated to be 4.62 million in 2012 and is projected to reach about 7 million by 2025, affecting nearly 1 in 5 people over the age of 65 years [6]. People with dementia experience memory loss and poor judgment and may take longer to complete normal daily tasks. Thus, the providers of medical and dental care cannot ignore problems associated with dementia. In the field of maxillofacial prosthetic treatment in particular, the current status of the care system must be considered for several reasons. The patient population is elderly and aging [4]. In addition, the structure of maxillofacial prostheses is quite unique compared with general prostheses, and the oral cavity is sensitive and prone to bleeding, especially in maxillectomy patients. Sumita et al. reported a case in which a maxillectomy patient experienced difficulty inserting his maxillary obturator prosthesis after developing cerebrovascular disease and dementia [3]. However, there has been no report on the actual situation of patients who need maxillofacial prosthetic treatment from the perspective of dementia and patient-reported difficulty of inserting prostheses. To clarify the issues in providing supportive care, including terminal care, to patients with maxillofacial defects, it is important to determine the prevalence of dementia and the factors associated with patients’ difficulties in inserting maxillofacial prostheses.

Many scales for dementia screening are available, including the clinical dementia rating (CDR), mini-mental state examination (MMSE), Montreal cognitive assessment (MoCA), Addenbrooke’s cognitive examination-III (ACE-III), and the revised Hasegawa’s dementia scale (HDS-R). The CDR has high validity and reliability for this purpose but requires a considerable amount of data from both the patient as well as an informant [7]. The MMSE is the most widely used screening method for cognitive impairment, including dementia, but its level of sensitivity and specificity for dementia has been criticized because it was originally developed to evaluate elderly psychiatric patients rather than people with dementia [8,9]. The MoCA-J is the Japanese translation of the original English version of the MoCA and was translated by a Japanese geriatrician and a Japanese psychologist. The MoCA-J was finalized after a pilot study involving 20 elderly volunteers. The diagnostic accuracy of the MoCA-J for detecting Alzheimer’s disease in healthy individuals (100% sensitivity and 89% specificity) was found to be superior to that of the MMSE (97% and 89%, respectively) but slightly inferior to that of the HDS-R (97% and 97%, respectively) [10]. The ACE-III is a screening technique that can differentiate patients with and without cognitive impairment and shows high sensitivity for the early stages of dementia [11]. Its subdomains are significantly correlated with neuropsychological tests specific to the domains assessed [11]. However, ACE-III has 100 items and takes 20 min to complete [11]. The HDS-R is the revised version of the HDS, a brief cognitive scale developed by Hasegawa in 1974 to screen for dementia [12]. The HDS-R is widely used both in the clinical setting and in epidemiological research in Japan and was developed after considering its feasibility for worldwide use [12]. It examines the main areas of cognitive function, including orientation, attention, language, and memory. Studies have shown that the HDS-R has high sensitivity for detecting Alzheimer’s disease in healthy individuals. The HDS-R is commonly used as a screening instrument in hospitals and welfare facilities for the elderly and in epidemiological surveys in Japan [13]. The scale can be completed in a short time (about 5 min) [14], has no movement-related or visual tasks, and is easy for healthy elderly people to complete. A study in the United Kingdom showed an increased incidence of dementia with age and the apparent influence of sex in Alzheimer’s disease [15]. Another study showed that Alzheimer’s disease and other dementias disproportionately affect women [16]. However, the applicability of these findings in elderly people with head and neck defects is not known.

Maxillofacial prostheses are an important tool in providing continuous supportive care to patients with maxillofacial defects, including in terminal care [1,2]. In other words, many patients would have difficulty surviving in daily life without their maxillofacial prosthesis [3]. Thus, ongoing care to enable their continuous use of the prosthesis is important. However, there has been no report focusing on patient-reported problems in inserting an obturator after maxillectomy. Additionally, patients’ approaches for overcoming these problems have not been shared. Thus, it is necessary to collect patients’ comments, opinions, and concerns about obturator insertion in order to provide a suitable clinical flow and effective care and to give appropriate advice to those who have difficulty inserting their obturator.

To provide supportive care for maxillectomy patients, it is necessary to analyze which factors may cause difficulty with inserting maxillary obturator prostheses. Insertion difficulty can be objectively reflected by the time taken to insert the prosthesis. The main factor expected to be related to insertion difficulty is the structure of the prosthesis and the cognitive and physical functioning of the patient. Structural factors for maxillary obturator prostheses include their weight, height, and clasps. We speculated that with increasing obturator height, the higher number of clasps would lead to greater weight and would increase the difficulties that patients experienced during prosthesis insertion. For patient factors, on the other hand, physical function, manual dexterity, coordination, strength, and cognitive function all decline with age [17], and we therefore speculated that these declines would increase the difficulties patients experience when inserting their prosthesis.

The aim of this study was to determine the prevalence of possible dementia at a maxillofacial prosthetic clinic in order to collect the patients’ subjective comments about the difficulty of inserting their maxillofacial obturator prosthesis and to reveal the factors associated with such difficulty after maxillectomy.

## 2. Materials and Methods

Informed consent was obtained before participation in the study, which was approved by the Ethics Committee of the Faculty of Dentistry, Tokyo Medical and Dental University (approval number D2016-012).

### 2.1. Participants

In part I of this study, patients aged 65 years or older who attended the maxillofacial prosthetics clinic at the Tokyo Medical and Dental University Dental Hospital between 2016 and 2022 were recruited, and 183 patients (100 men, and 83 women) agreed to participate in our survey regarding the possible prevalence of dementia.

In part II, 56 of the 183 patients who participated in the survey on dementia who had a maxillary defect and used a maxilla obturator prosthesis after maxillectomy were recruited between October 2020 and November 2022. Nine of the participants discontinued treatment for personal reasons and were excluded from the analysis. The remaining 47 patients (23 men, 24 women) completed a questionnaire and five assessments: the HDS-R, the Purdue Pegboard Test (PPT), maxillary obturator prosthesis insertion time, grip strength, and the understanding of the intraoral surgical site. In some cases, tests were completed over two visits to minimize the burden on the patient and prevent fatigue from affecting the results.

### 2.2. Part I: Survey on the Prevalence of Possible Dementia

Participants completed the HDS-R in a face-to-face setting (Table 1). The nine items of the HDS-R are as follows: item 1 concerns age (1 point) and tests self-orientation; item 2 concerns the date (4 points) and tests the relationship to time; item 3 concerns the location (2 points) and tests orientation to place; item 4 is the repetition of three familiar words (3 points), which tests the immediate memory; item 5 involves the subtraction of 7s from 100 (2 points), which assesses computational ability, immediate memory, and working memory; item 6 involves the backward repetition of three and four digits (2 points), which tests working memory; item 7 involves the recall of the three words out of four (6 points), which tests recent memory; item 8 involves the immediate recall of five objects that are shown then hidden (5 points), which tests the immediate memory and visual memory; and item 9 involves the listing of 10 vegetable names (5 points) tests language fluency [12,18]. The maximum score on the HDS-R is 30 points. A score of 20 or less is indicative of possible dementia, and a score of 10 or less is indicative of severe dementia.

### 2.3. Part II: Difficulty Inserting a Maxillary Obturator Prosthesis after Maxillectomy

#### 2.3.1. Questionnaire

Participants completed a three-part questionnaire. The first part was a subjective evaluation by the patients concerning the difficulties they experienced when inserting their maxillary obturator prosthesis, as measured on a visual analogue scale (VAS 0–100). The second part asked them for personal comments regarding the cause of the insertion problems. They were also asked to share any personal advice for inserting a maxillary obturator prosthesis. The third part asked whether they had worn normal dentures before the maxillectomy and for how long.

#### 2.3.2. Basic Information on Intraoral Condition and Maxillary Obturator Prostheses

The intraoral data collected included whether there was communication between the oral and nasal cavities due to the surgical defect, the number of remaining teeth in the maxilla, and the degree of mouth opening. The information collected about the maxillary obturator prosthesis included the material, weight, obturator height, and number of clasps.

#### 2.3.3. HDS-R

The HDS-R was used to assess cognitive function. A score of ≤20 was taken to indicate possible dementia [14].

#### 2.3.4. PPT

The PPT was used to evaluate manual dexterity and coordination in both hands [19]. Participants were seated comfortably at the testing table with the pegboard on the table in front of them. The pegboard had 4 cups across the top and two vertical rows of 25 small holes down the center. The four cups contained 25 pins each. This test measured how many pins could be inserted into a row in 30 s with each hand. The test was performed twice for each hand and the highest score of the dominant hand was used for analysis.

#### 2.3.5. Maxillary Obturator Prosthesis Insertion Time

As an outcome for assessing the difficulty of inserting a maxillary obturator prosthesis, the time required for insertion was measured from when participants picked up their prosthesis from the table to when they raised their hand after it was in place. After the test, we checked whether the prosthesis was fully and correctly in place and, if so, we recorded the insertion time.

#### 2.3.6. Grip Strength Test

There is no internationally unified standard for grip strength testing, and opinions differ on whether the results are consistent between measurements taken in the standing and sitting positions [20]. Considering the ease of testing, grip strength was measured with an electronic hand dynamometer (N-FORCE, Wakayama, Japan) in the left and right hands while the participants were standing with the arms hanging down naturally. Data from the dominant hand were used.

#### 2.3.7. Understanding of the Intraoral Surgical Site

Participants were asked to circle the surgical site on an intraoral schematic drawing of the upper and lower dentition.

### 2.4. Statistical Analysis

#### 2.4.1. Part I: Prevalence of Possible Dementia

Because the total score on the HDS-R was not normally distributed (Shapiro–Wilk test, *p* < 0.001), a two-sided Mann–Whitney U test was used for comparisons by sex. Frequency analysis was performed using Fisher’s exact test. A *p*-value of less than 0.05 was considered to indicate statistical significance. All statistical analyses were performed using SPSS statistics version 28 (IBM Corp., Armonk, NY, USA).

#### 2.4.2. Part II: Difficulty Inserting the Maxillary Obturator Prosthesis after Maxillectomy

To reveal factors associated with the difficulty of inserting a maxillary obturator prosthesis, the insertion time was selected as an outcome measure for difficulty. Multiple regression analysis was used to identify factors associated with the insertion time. All variables were entered into the multiple regression model. A *p* value of less than 0.05 was considered to indicate statistical significance. Statistical analysis was performed using SPSS statistical ver. 28 (IBM Corp.).

## 3. Results

### 3.1. Part I: Prevalence of Possible Dementia

Among the 183 participants, HDS-R scores were ≤20 in 16 and ≤10 in 3, giving a prevalence of possible dementia of 8.7% (95% CI: 4.6–12.9%). The HDS-R score was ≤20 in 12% of the male participants but 4.8% of the female participants.

**Figure 1:**
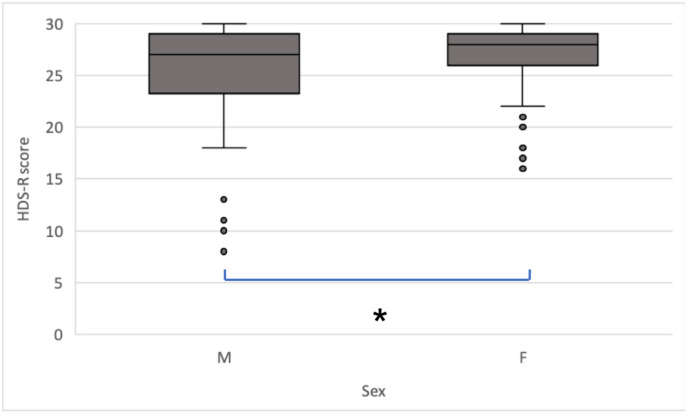
HDS-R scores by sex. Scores were significantly higher in women than in men (* *p* = 0.037, two-sided Mann–Whitney U test). The center line shows the mean, boxes show the IQR, and whiskers indicate the range. Dots indicate outliers.

Median age was 78 (interquartile range [IQR]: 71–82) years in men and 77 (IQR: 72–84) years in women. HDS-R score between men and women showed a significant difference (*p* < 0.05, Mann–Whitney U test) (Figure 1).

In this study, following the definition of old age used by the United Nations and WHO (≥65 years), the 183 patients were divided into three age groups: 65–74 years (n = 71), 75–84 years (n = 80), and ≥85 years (n = 32). As shown in Table 2, there were significant differences in HDS-R total score between the 65–74-year age group and the 75–84-year age group and between the 65–74-year age group and the ≥85-year age group (*p* < 0.05, Fisher’s exact test).

**Figure 2:**
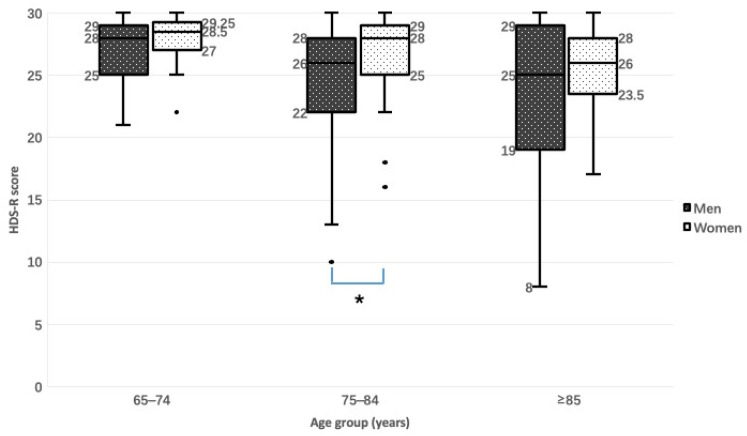
Relationship between HDS-R score and sex by age group. * *p* = 0.044, two-sided Mann–Whitney U test.

There was a significant difference in HDS-R total score between men and women in the 75–84-year age group according to the two-sided Mann–Whitney U test (Figure 2). HDS-R score decreased with age in men and in women.

Table 3 shows scores for each HDS-R item for men, women, and all participants. Sex differences were seen in the HDS-R item scores for working memory (item 6), recent memory (item 7), and language fluency (item 9). The complete accuracy rate was low for recent memory (item 7) at 41.5% (95% CI: 34.3–48.7%) compared with the other items.

### 3.2. Part II: Difficulty Inserting a Maxillary Obturator Prosthesis after Maxillectomy

Of the 47 participants asked for subjective comments about insertion difficulties, 26 responded that they had such a difficulty. In particular, 12 indicated the difficulty was due to an oral defect, 5 due to limited mouth opening, and 4 due to hand coordination problems. Regarding the participants’ advice for inserting maxillofacial obturator prostheses, 14 participants said to follow the doctor’s instructions and practice repeatedly in order to gradually become accustomed to inserting the prosthesis. One participant responded that he used his tongue to assist with insertion. The other participants did not provide advice (Table 4).

Table 5 shows the intraoral data, information about the maxillary obturator prostheses, and results of the five assessments for each participant.

Multiple regression analysis identified which of the factors we had hypothesized would affect prosthesis insertion time (Table 6). Not all of these factors had an impact on the insertion time. Age, number of remaining teeth, number of clasps, grip strength, and understanding of the intraoral surgical site showed higher VIF scores.

**Figure 3:**
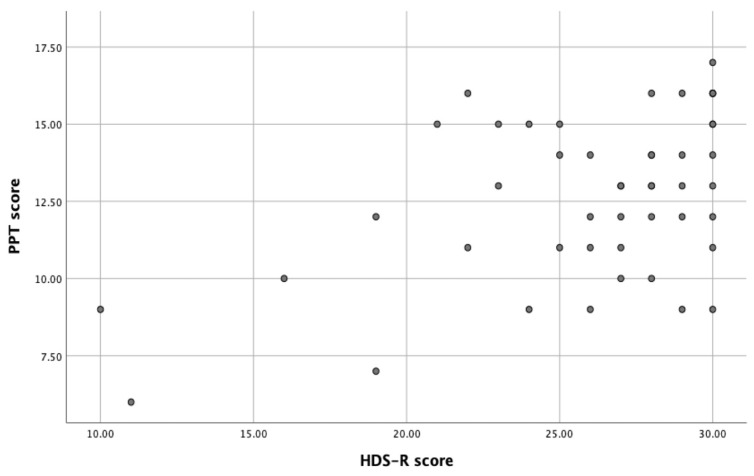
Correlation between HDS-R score and PPT score.

Communication between the oral and nasal cavities, number of remaining teeth, prothesis material, number of clasps, and obturator height can affect the weight of the maxillary obturator prosthesis. The HDS-R score was related to age and sex according to the results of part I in this study. The HDS-R item concerning the understanding of self-information and concerning understanding of intraoral surgical site were related. The correlation between HDS-R score and PPT score was significant (*p* < 0.05, Spearman’s rank correlation test) (Figure 3). Grip strength was related to age and sex, as reported previously [17].

After trying different models and comparing the adjusted R^2^ value among the models, multiple regression analysis identified the affecting factors of prosthesis insertion time (Table 7, Model 2). Multicollinearity was assessed using VIF scores. Because age was always considered as an affecting factor, it was added to Model 1. Without age, the VIF score was decreased when comparing Model 1 and Model 2. Both models showed that HDS-R score had a significant impact on the prosthesis insertion time in patients with maxillectomy. The *p*-values for duration of wearing dentures before maxillectomy and grip strength were close to 0.05 in the second model, so these two factors have an impact on the prosthesis insertion time in patients with maxillectomy.

## 4. Discussion

From our survey, 8.7% of patients who visited our maxillofacial prosthetics clinic at a university hospital between 2016 and 2022 were found to have possible dementia according to the HDS-R. This corresponds to about 1 in 12 elderly patients. After dividing the patients into three age groups, namely 65–74 years (n = 71), 75–84 years (n = 80), and ≥85 years (n = 32), significant differences in the HDS-R score were found between youngest age group and each of the two older age groups, indicating that the prevalence of possible dementia increased with age in our patients. However, there was no difference between the intermediate age group and the oldest group. This suggests that patients over 75 years old require more attention and their cognitive and physical condition should be monitored in order to detect changes in their status and to provide more support when needed.

There was a significant difference in the prevalence of possible dementia between men and women. Compared with female patients, more male patients had an HDS-R score of ≤20, particularly ≤10. This result is different from a previous study that found women are more likely to develop dementia [16]. These conflicting results might have arisen from a difference in the method for evaluating dementia. Another possibility is that the characteristics of patients with head and neck defects have some influence on the prevalence of dementia, and follow-up research is needed to focus on this aspect.

Sex differences were noted in the HDS-R total score and individual item scores (Table 3). In this study, men’s scores for item 6 were higher than women’s, but women’s scores for items 7 and 9 were higher than men’s. This could suggest that men’s immediate memory and visual memory are better than women’s, but women’s recent memory and language fluency are better than men’s. This might be related to men’s and women’s family life roles for this generation of patients in Japan, where men were more likely to take on a work role and women were more likely to take on a domestic role. This might account for women’s better recent memory and the recall of vegetable names. This result suggests that when a high-frequency behavior occurs in life, the things involved in the behavior are easily recalled. The item 7 scores for recent memory were lower scores than the other item scores. This result is consistent with the fact that older people tend to easily forget recent events.

From the maxillectomy patients’ subjective comments about difficulties inserting their maxillofacial obturator prosthesis (Table 4), it was revealed that 34% of them found insertion slightly difficult and 55% experienced clear problems when inserting them. According to the patients’ comments regarding the reason for the difficulty, 26% attributed it to their intraoral defect, while 10% and 9% attributed it to a limitation of mouth opening and hand coordination problems, respectively. Thus, when providing continuous care for these patients, we should proactively ask patients about potential difficulties they might be experiencing, bearing in mind these three main reasons.

From the results of Table 7, we can see that the HDS-R score had a significant impact on difficulty inserting a maxillary obturator prosthesis. Moreover, age, the duration of wearing dentures before maxillectomy, the prosthesis weight, and grip strength were related to such difficulty. The duration of wearing dentures before maxillectomy was associated with increased difficulty, which may be related to the different ways that dentures and maxillary obturator prostheses are inserted and to the age of the patients. Because the duration of wearing dentures previously did not seem to help the patients with insertion, it seems that the oral defect itself also likely increased the difficulty. The weight of the maxillary obturator prosthesis depends on its structure, suggesting its structure could also have an impact on the difficulty experienced by patients. We initially thought that the weight was related to the defect area; however, it could also be related to many other factors, such as material differences and number of clasps. Thus, it is unclear which is the most important factor. The HDS-R score was related to the PPT score, indicating that cognitive decline was accompanied by declines in manual dexterity and coordination. In addition, age, HDS-R score, and grip strength were associated with increased insertion difficulty. Therefore, both cognitive and physical decline, but especially cognitive decline, can contribute to patients’ difficulties inserting their maxillary obturator prosthesis.

When we combine the comments we collected from patients with the results of our analysis, we consistently find that prosthesis structure and decreased hand manual dexterity and coordination were related to increased insertion difficulty. When we combine the conclusions of the two parts of this study, we find that declines in cognitive function, manual dexterity, and grip strength were related to increased insertion difficulty, and these functional declines are related to age. Therefore, in the treatment of older patients, it is necessary to assess their cognitive ability and hand function in a timely manner. If functional decline is evident, the structure of the maxillary obturator prosthesis should be adjusted promptly to make it easier for them to wear, and more attention and support should be given. Then, when we combine the advice from patients with our two conclusions, it seems that things and events involving high-frequency behaviors earlier in life are relatively easily recalled by older people while recent events are more easily forgotten. To reduce the difficulty of inserting a maxillary obturator prosthesis, older patients may need repeated reminding and guidance as well as increased practice.

Based on the above conclusions, for maxillectomy patients who have difficulty inserting their maxillary obturator prosthesis, the first step is to communicate with the patients to understand the nature of the difficulty and to determine which part of the prosthesis design is connected with the problem. After addressing any design issues, in order to lessen the difficulty experienced by these patients, they need to regularly practice inserting their prosthesis to form a habit. If they wore dentures before the maxillectomy, it would be helpful to highlight the differences between wearing a maxillary obturator prosthesis and dentures for them and encourage them to practice insertion. In patients with severely impaired cognitive and hand functions, prompt communication with the patient’s family members or caregivers is necessary, so that they can help the patient safely and correctly insert and use their maxillary obturator prosthesis.

A previous study found that implants with appropriate attachment systems dramatically improved the retention of the maxillary obturators [21], which may have an impact on patients’ insertion of maxillary obturator prostheses. At present, there are not many elderly patients with implants in our clinic, and none of the participants in this study had implants. As the use of implants increases, we will pay attention to whether they increase the difficulty in prothesis insertion in follow-up studies.

There are some limitations to this study. The first is that the HDS-R was used for the screening of possible dementia. The HDS-R was developed after considering its feasibility for worldwide use [12], but it does not have a drawing and reading component, making it impossible to test these abilities in older adults with hearing impairments. Therefore, future research is needed to identify the clinical population with possible dementia by using other scales, such as the widely used MMSE. The second limitation is the small number of patients with dementia in this study. This may be due to the characteristics of our clinic, which belongs to a university hospital; is located in a bustling area of Tokyo, Japan; and has a large train station nearby. These may all be factors affecting the patients who visit our clinic. The third limitation is that maxillary obturator prosthesis insertion time was selected as an outcome measure for the difficulty of inserting the maxillary obturator. The insertion time may be affected by factors such as the patient’s personality, so a better outcome measure for this is needed. Lastly, the structure of maxillary obturator prostheses is complicated and the information collected was not sufficient to analyze which part of the structure was connected with patients’ difficulties with insertion. Future studies could investigate additional information, such as defect location and defect area, and then group and compare the prosthesis structures. In terms of patients practicing inserting their maxillary obturator prosthesis, virtual reality (VR) that gamifies practice could be one solution.

The results of this study indicate issues to consider for continuous medical and dental care in a super-aging society. The possibilities of patients having dementia and/or difficulties inserting their maxillofacial prosthesis should be kept in mind when providing supportive care, including terminal care, for patients with maxillary defects.

## 5. Conclusions

In this study, 8.7% of the participants were judged to have possible dementia, and a lower HDS-R score had a significant impact on insertion difficulty. Cognitive function and difficulty inserting maxillary obturator prostheses should be considered in the provision of continuous supportive care to patients with maxillary defects.

## Figures and Tables

**Table 1:** The revised Hasegawa’s dementia scale.

Item No.	Questions		Score
1	Age (1 point if correct within ±2 years)		0, 1
2	Year, month, date, day (1 point each)	Year Month Date Day	0, 1 0, 1 0, 1 0, 1
3	Current location (2 points for a correct answer given within 5 s; 1 point for correct choice between hospital and office)		0, 1, 2
4	Repeating 3 words (1 point each; use only one version per test) 1 (a) cherry blossom, (b) cat, (c) train; 2 (a) plum blossom, (b) dog, (c) car		0, 1 0, 1 0, 1
5	100 − 7 = ? (1 point for the correct answer; if not correct, skip to item 6) −7 again = ? (1 point for a correct answer)	(93) (86)	0, 1 0, 1
6	Repeating 6-8-2 backward (Skip to item 7 if not correct answer) Repeating 3-5-2-9 backward	2-8-6 9-2-5-3	0, 1 0, 1
7	Recalling 3 words from item 4. (2 points for spontaneous recall; 1 point for a correct recall after category cue) (a) plant, (b) animal, (c) transportation		a: 0, 1, 2 b: 0, 1, 2 c: 0, 1, 2
8	Recalling 5 unrelated common objects after they are shown and then covered (Scissors, mirror, battery, pen, coin, etc.)		0, 1, 2 3, 4, 5
9	Naming all vegetables that come to mind (Stop after a 10 s interval with no response) 0–5 = 0 points, 6 = 1 point, 7 = 2 points, 8 = 3 points, 9 = 4 points, 10 = 5 points		0, 1, 2 3, 4, 5
Total score			

**Table 2:** Differences in total score on the HDS-R according to age group.

Age Group (Years)	HDS-R Score		Adjusted *p*-Value
	0–20 ^#^	21–30	
65–74	0	71	65–74 vs. 75–84: 0.010 *
75–84	9	71	65–74 vs. ≥85: 0.001 *
≥85	7	25	75–84 vs. ≥85: 0.690
Total	16	167	

^#^: HDS-R score ≤ 20 indicates possible dementia. *: Significant difference between age groups (adjusted *p* < 0.05, Fisher’s exact test with Bonferroni correction for multiple comparisons).

**Table 3:** Sex differences for total score and individual item score on the HDS-R.

Question	Score	Male, n (%)	Female, n (%)	Total, n (%)	*p*-Value
1 (Age)	0	2 (2)	0 (0)	2 (1.1)	0.502
	1	98 (98)	83 (100)	181 (98.9)	
2 (Date)	0–3	13 (13)	11 (13.3)	24 (13.1)	1
	4	87 (87)	72 (86.7)	159 (86.9)	
3 (Place)	0–1	2 (2)	0 (0)	2 (1.1)	0.502
	2	98 (98)	83 (100)	181 (98.9)	
4 (Repetition)	0–2	1 (1)	0 (0)	1 (0.5)	1
	3	99 (99)	83 (100)	182 (99.5)	
5 (Subtraction)	0–1	15 (15)	17 (20.5)	32 (17.5)	0.337
	2	85 (85)	66 (79.5)	151 (82.5)	
6 (Backward repetition)	0–1	23 (23)	32 (38.6)	55 (30.1)	0.024 *
	2	77 (77)	51 (61.4)	128 (69.9)	
7 (Recall)	0–5	66 (66)	41 (49.4)	107 (58.5)	0.025 *
	6	34 (34)	42 (50.6)	76 (41.5)	
8 (Naming objects)	0–4	44 (44)	28 (33.7)	72 (39.3)	0.174
	5	56 (56)	55 (66.3)	111 (60.7)	
9 (Naming vegetables)	0	14 (14)	0 (0)	14 (7.7)	<0.001 **
	1–2	17 (17)	5 (6)	22 (12)	
	3–4	9 (9)	13 (15.7)	22 (12)	
	5	60 (60)	65 (78.3)	125 (68.3)	
Total score	0–10	3 (3)	0 (0)	3 (1.6)	0.178
	11–20	9 (9)	4 (4.8)	13 (7.1)	
	21–30	88 (88)	79 (95.2)	167 (91.3)	

* Men vs. women: *p* < 0.05, ** *p* < 0.01, Fisher’s exact test.

**Table 4:** Comments from the participants.

Subjective Comments	(N = 47)
Visual analogue scale score	n (%)
<50	1 (2)
≥50, <70	4 (9)
≥70, <90	11 (23)
≥90, <100	31 (66)
Problems with inserting a maxillary obturator prosthesis	n (%)
No	21 (45)
Yes	26 (55)
Reasons for problems	
Defect	12 (26)
Hand coordination	4 (9)
Mouth opening	5 (10)
Other	5 (10)
Advice for inserting a maxillary obturator prosthesis	n (%)
Follow doctor’s instructions and become accustomed to it	14 (30)
Use tongue to help	1 (2)
No advice provided	32 (68)

**Table 5:** Characteristics of the participants.

Characteristics	(N = 47)
Age (years), (mean ± SD)	74 ± 8
Sex, n	
Men	23
Women	24
Duration of wearing dentures before maxillectomy (year), median (range, IQR)	0 (0–40, 7)
Communication between the oral cavity and nasal cavity, n	
Yes	31
No	16
Number of remaining teeth, median (range, IQR)	3 (0–10, 6)
Mouth opening (mm), median (range, IQR)	42 (11–70, 14)
Prosthesis material, n	
Resin base denture	43
Metal flame denture	4
Prosthesis weight (g) (mean ± SD)	25.8 ± 10.4
Obturator height (mm) (mean ± SD)	24.4 ± 11.2
Number of clasps, median (range, IQR)	2 (0–6, 3)
HDS-R total score, median (range, IQR)	27 (10–30, 5)
PPT score (mean ± SD)	13 ± 3
Insertion time, median (range, IQR)	5 (2–31, 3.39)
Grip strength (kg), median (range, IQR)	24.5 (13.9–48.2, 13)
Understanding of the surgical site, n	
Yes	39
No	8

**Table 6:** Results of multiple regression analysis for all factors hypothesized to affect prosthesis insertion time in patients with maxillectomy.

Potential Affecting Factors	Regression Coefficient		Standardized Coefficients	*p* Value	VIF
	B	Std. Error			
Age	0.080	0.167	0.130	0.634	3.875
sex	−2.359	2.394	−0.244	0.332	3.255
Duration of wearing dentures before maxillectomy (years)	0.114	0.102	0.203	0.272	1.758
Communication between the oral and nasal cavities	0.923	1.555	0.090	0.557	1.234
Number of remaining teeth	0.048	0.387	0.034	0.901	3.992
Degree of mouth opening	−0.032	0.068	−0.077	0.643	1.458
Prosthesis materials	−1.356	2.916	−0.078	0.645	1.504
Prosthesis weight	−0.027	0.085	−0.057	0.755	1.745
Obturator height	−0.013	0.079	−0.030	0.870	1.718
Number of clasps	−0.328	0.820	−0.110	0.692	3.997
HDS-R total score	−0.394	0.251	−0.379	0.127	3.113
PPT score	−0.102	0.462	−0.054	0.827	3.213
Grip strength	−0.216	0.155	−0.383	0.174	4.036
Understanding the surgical site	−1.937	3.572	−0.150	0.591	4.095

Adjusted R^2^: 0.136 (*p* > 0.05).

**Table 7:** Affecting factors for prosthesis insertion time in patients with maxillectomy.

**(a) Model 1**					
**Affecting Factors**	**Regression Coefficient**		**Standardized Coefficients**	***p* Value**	**VIF**
	**B**	**Std. Error**			
**Age**	**0.112**	**0.105**	0.182	0.289	1.79
Duration of wearing dentures before maxillectomy (years)	0.135	0.079	0.24	0.102	1.292
Prosthesis weight	−0.013	0.061	−0.029	0.828	1.06
HDS-R score	−0.357	0.157	−0.344	0.028	1.43
Grip strength	−0.110	0.092	−0.194	0.24	1.662
Adjusted R^2^: 0.264					
**(b) Model 2**					
**Affecting Factors**	**Regression Coefficient**		**Standardized Coefficients**	***p* Value**	**VIF**
	**B**	**Std. Error**			
**Duration of wearing dentures before maxillectomy (years)**	**0.154**	**0.079**	0.274	0.058	1.232
Prosthesis weight	−0.025	0.06	−0.053	0.68	1.026
HDS-R score	−0.437	0.139	−0.420	0.003	1.112
Grip strength	−0.154	0.082	−0.273	0.069	1.328
Adjusted R^2^: 0.261					

## Data Availability

Data sharing not applicable.
